# Innovative Programs in Aging

**DOI:** 10.1093/geroni/igaf122.1771

**Published:** 2025-12-31

## Abstract

To address the increasing complexity in care of older adults, frameworks and innovative programs will be presented during this session.

